# Supplementary material for: (20S) Ginsenoside Rh2 Inhibits STAT3/VEGF Signaling by Targeting Annexin A2
Source: Int J Mol Sci. 2021 Aug 27;22(17):9289. doi: 10.3390/ijms22179289 (PMC8431727; doi:10.3390/ijms22179289)
Supplement: Supplementary file 1 [file ijms-22-09289-s001.zip › ijms-1307037-supplementary.pdf]

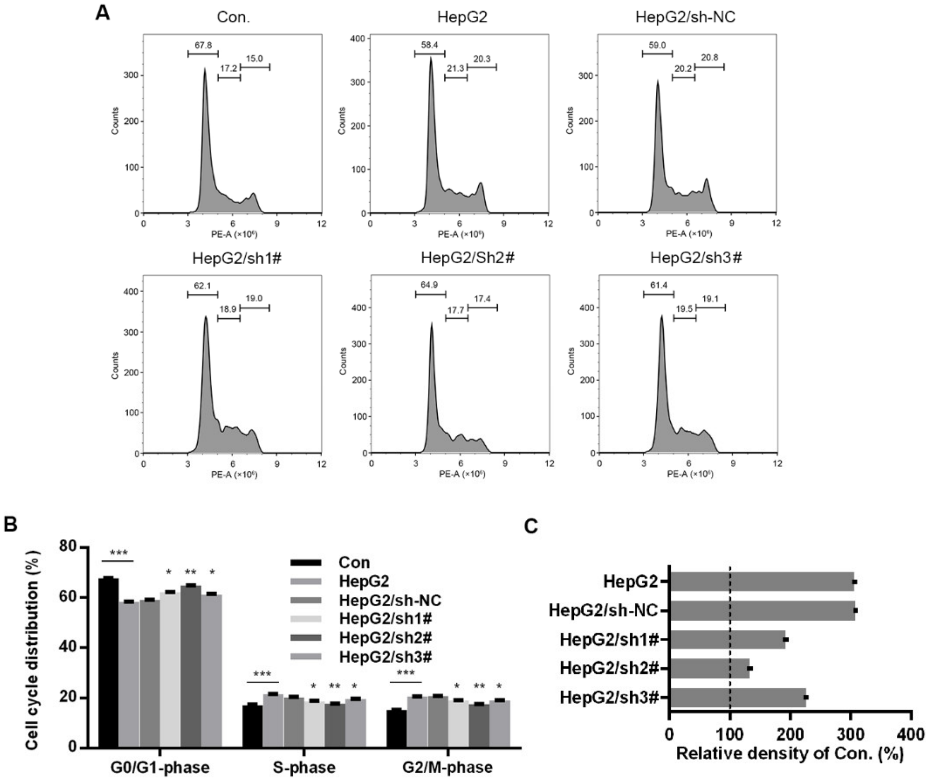

Figure S1. (**A**, **B**) The cell cycle of HUVECs co-cultured with HepG2 cells was determined by flow-cytometry. The florescence distribution (**A**) of HUVECs and cell cycle distribution (**B**) were shown as histogram. (**C**) Quantitative analysis of migrated HUVECs attached to Fig. 2F.

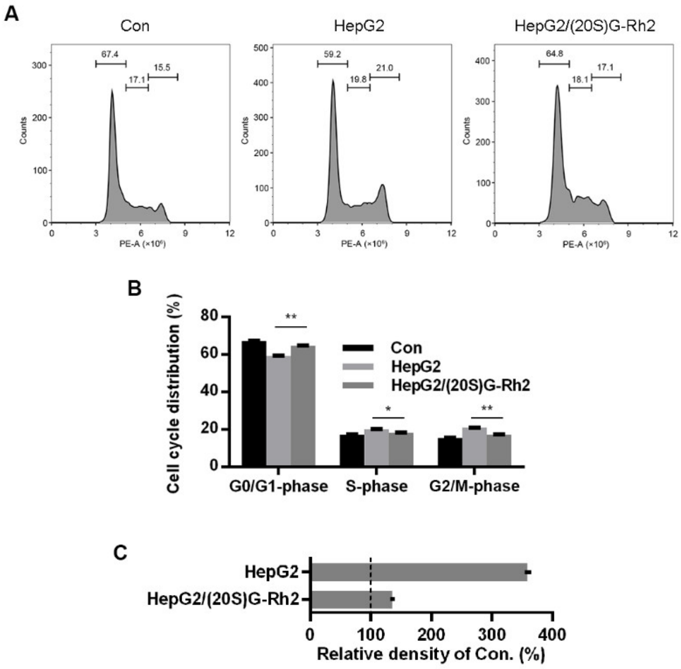

Figure S2. (**A**, **B**) The cell cycle of HUVECs co-cultured with (20S)G-Rh2 pre-treated HepG2 cells was determined by flow-cytometry. The florescence distribution (**A**) of HUVECs and cell cycle distribution (**B**) were shown as histogram. (**C**) Quantitative analysis of migrated HUVECs attached to Fig. 4F.

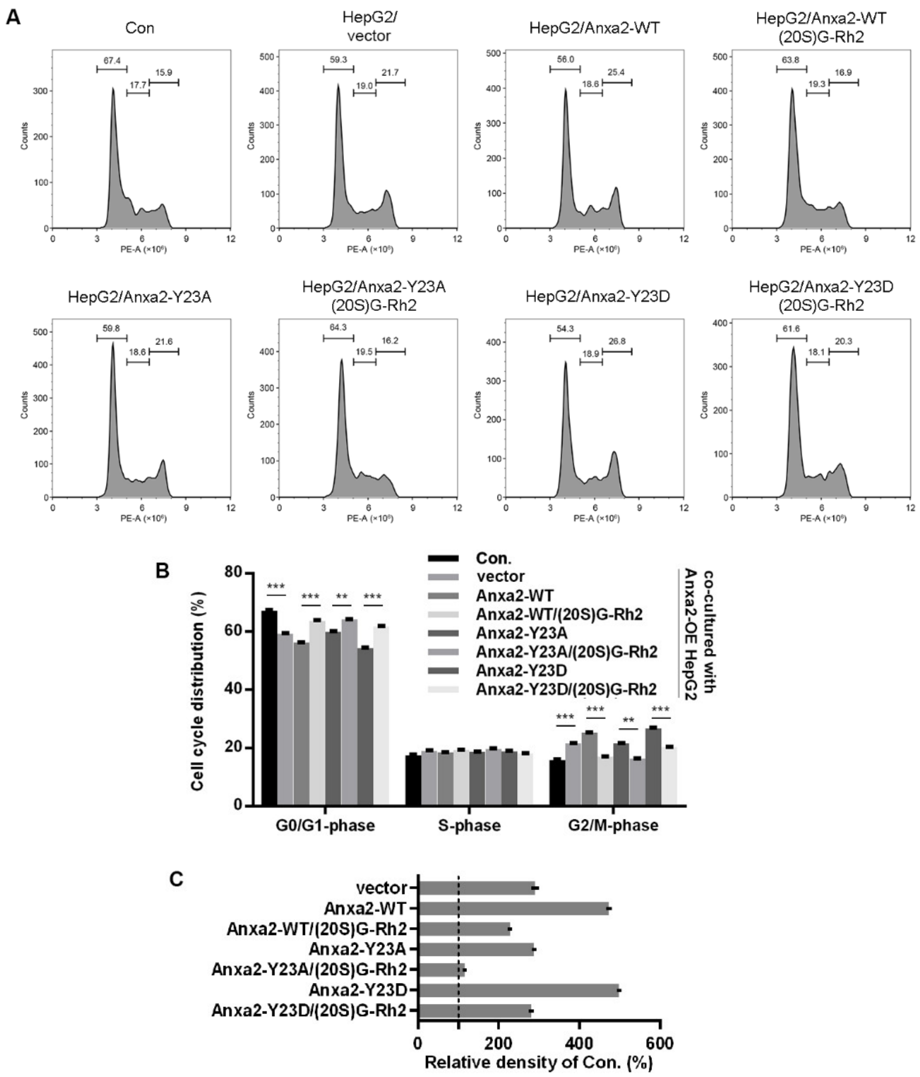

Figure S3. (20S)G-Rh2 inhibited Anxa2-Y23D mutant-enhanced proliferation and migration of HUVECs. (**A**, **B**) The cell cycle of HUVECs co-cultured with Anxa2-over-expressing HepG2 cells was determined by flow-cytometry. The florescence distribution (**A**) of HUVECs and cell cycle distribution (**B**) were shown as histogram. (**C**) Quantitative analysis of migrated HUVECs attached to Fig. 6F.

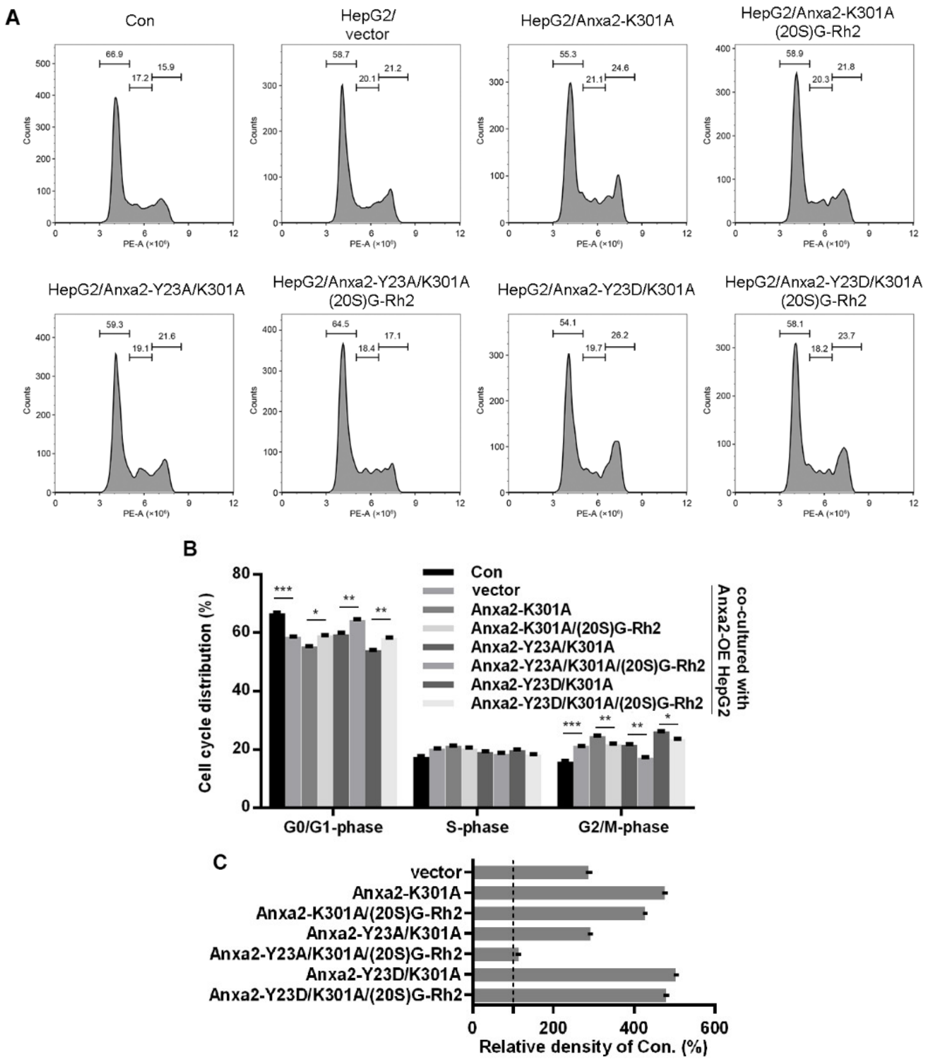

Figure S4. Anxa2-K301A mutant maintained pro-proliferation and pro-migration capability of HepG2 cells under (20S)G-Rh2 treatment. (**A**, **B**) The cell cycle of HUVECs co-cultured with Anxa2-over-expressing HepG2 cells was determined by flow-cytometry. The florescence distribution (**A**) of HUVECs and cell cycle distribution (**B**) were shown as histogram. (**C**) Quantitative analysis of migrated HUVECs attached to Fig. 8F.

Table S1 Sequences of Anxa2 shRNA vectors

No.		Sequences
1# Anxa2-Homo-741	Target	GATAGGTACAAGAGTTACAGC
	Sense	CACCGATAGGTACAAGAGTTACAGCTTCAAGAGAGCTGTAACTCTTGTACCTATCTTTTTTG
	Anit-sense	GATCCAAAAAAGATAGGTACAAGAGTTACAGCTCTCTTGAAGCTGTAACTCTTGTACCTATC
2# Anxa2-Homo-967	Target	CTGAATTCAAGAGAAAGTACG
	Sense	CACCGCTGAATTCAAGAGAAAGTACGTTCAAGAGACGTACTTTCTCTTGAATTCAGTTTTTTG
	Anit-sense	GATCCAAAAAACTGAATTCAAGAGAAAGTACGTCTCTTGAACGTACTTTCTCTTGAATTCAGC
3# Anxa2-Homo-1116	Target	CTTCCAGCTAACAGGTCTAGA
	Sense	CACCGCTTCCAGCTAACAGGTCTAGATTCAAGAGATCTAGACCTGTTAGCTGGAAGTTTTTTG
	Anit-sense	GATCCAAAAAACTTCCAGCTAACAGGTCTAGATCTCTTGAATCTAGACCTGTTAGCTGGAAGC

Table S2 Primer list of STAT3 target genes

Gene Name		Sequences
CDC25A	F	TCTGGACAGCTCCTCTCGTCAT
	R	ACTTCCAGGTGGAGACTCCTCT
CDC25C	F	AGAAGCCCATCGTCCCTTTGGA
	R	GCAGGATACTGGTTCAGAGACC
CDC37B	F	GAGAGTGACTTCGACGTGTTCC
	R	TCCATTTGCACGCCACAGAGCT
CCND1	F	TCTACACCGACAACTCCATCCG
	R	TCTGGCATTTTGGAGAGGAAGTG
CCNG1	F	CCTTCTGTGTTGGCATTGTCTATC
	R	CAAGCTCTTGCCAGAAGGTCAG
CCNI	F	CTTCACACAGCCACACCATTGG
	R	GTAGTTGCTTGGTAAGGACTGCC
CCNJ1	F	ACTGGACCTGTTTATGGACCGC
	R	TGCTCCAGCTTAGGCACACTGT
CCNK1	F	CACTATGATACCCTGGCAACTGG
	R	CAGAAAGAGGCAACAGGCTCCT
EGFR	F	AACACCCTGGTCTGGAAGTACG
	R	TCGTTGGACAGCCTTCAAGACC
VEGFA	F	TTGCCTTGCTGCTCTACCTCCA
	R	GATGGCAGTAGCTGCGCTGATA
VEGFB	F	AAGGACAGTGCTGTGAAGCCAG
	R	TGGAGTGGGATGGGTGATGTCA
VEGFC	F	GCCAATCACACTTCCTGCCGAT
	R	AGGTCTTGTTCGCTGCCTGACA
VEGFD	F	GACTGGAAGCTGTGGAGATGCA
	R	GGCTGCACTGAGTTCTTTGCCA
PTEN	F	TGAGTTCCCTCAGCCGTTACCT

	R	GAGGTTTCCTCTGGTCCTGGTA
GAPDH	F	GTCTCCTCTGACTTCAACAGCG
	R	ACCACCCTGTTGCTGTAGCCAA
